# The Efficacy and Safety of Propranolol in Treating Infantile Hemangioma: A Prospective Study

**DOI:** 10.5812/ijpr-135140

**Published:** 2023-10-14

**Authors:** Mohammad Reza Khalilian, Fariba Esmaeili, Mohamad Reza Vahidi, Mohsen Rouzrokh, Elham Abdoulahzadeh, Hadi Pashapour, Mohammad Ghazavi

**Affiliations:** 1 Department of Pediatrics, School of Medicine, Shahid Beheshti University of Medical Sciences, Tehran, Iran; 2 Department of Medical Nanotechnology, School of Advanced Technologies in Medicine, Tehran University of Medical Sciences, Tehran, Iran; 3 Research Center of Mofid Children's Hospital, Shahid Beheshti University of Medical Sciences, Tehran, Iran; 4 Department of Epidemiology, School of Public Health & Safety, Shahid Beheshti University of Medical Sciences, Tehran, Iran; 5 Department of Pediatrics, Kashan University of Medical Sciences and Health Services, Kashan, Iran

**Keywords:** Infantile Hemangioma, Prospective, Descriptive, Propranolol

## Abstract

Hemangiomas are benign vascular tumors that often develop in infants. The most common treatment option for complicated hemangiomas is propranolol. We discuss the use of oral propranolol in treating infantile hemangioma (IH) in an Iranian population at our hospital. We conducted a cross-sectional prospective descriptive study on 62 infants aged 1 to 16 months from 2017 to 2021. Propranolol was gradually administered orally at a dose of 3 mg/kg/day. The hemangioma score was examined at 6 intervals (first visit, 1, 3, 6, 9, and 12 months later). Propranolol therapies were stopped when there was no further decrease in scores for 2 successive visits. The study was completed by 62 patients. In terms of hemangiomas, 46 (74.2%) patients had 1 lesion, 12 (19.4%) had 2 lesions, and 4 (6.5%) had 3 lesions. Over time, the average size of hemangiomas steadily decreased, such that 5 patients (9.1%) were completely treated; 1 patient improved after 3 months, 3 after 6 months, 1 after 9 months, and 57 (91.9%) were partially treated. Aside from being safe and effective, propanol can also obtain a higher response rate when treatment is started early in infants aged less than 3 months.

## 1. Background

Propranolol is a nonselective β-adrenergic receptor antagonist used to treat hypertension, ischemic heart disease, arrhythmias, heart failure, thyrotoxicosis, migraine, and glaucoma at a dose of 1 - 5 mg/kg/day (1, 2). After the first report on the success of this medication in treating hemangiomas in 2014, followed by large retrospective multicenter randomized clinical trials (RCTs) in Europe and the US in 2015, the US Food and Drug Administration (FDA) and the European Medicines Agency (EMA) approved oral propranolol hydrochloride solution for the systemic treatment of proliferation of hemangiomas (3, 4). The gold standard treatment for high-risk hemangioma proliferation is oral propranolol therapy. Propranolol hydrochloride is available in solution and tablet forms, and its recommended dosage for hemangiomas is 2 - 3 mg/kg/day divided into two doses; still, the best results are obtained with 3 mg/kg/day divided into two doses for at least 6 months and often up to 12 months, depending on the size and severity of the hemangioma and possible side effects (5). There is no clear mechanism of action determined for propranolol on hemangiomas; however, there is a hypothesized mechanism that involves vasoconstricting the blood vessels, inhibiting angiogenesis by lowering vascular endothelial growth factor (VEGF) and basic fibroblast growth factor (βFGF) levels, inhibiting nitric oxide production, and suppressing the renin-angiotensin system. There are rare but serious side effects of propranolol, including bradycardia (0.1%), hypotension (0.1%), hypoglycemia (0.6%), bronchospasm, and bronchial hypersensitivity (0.9% - 12.9%). In addition, propranolol can cause sleep disorders (2% - 18.5%), drowsiness, irritability, diarrhea, constipation, and cold extremities. However, these side effects are not an indication for discontinuing propranolol (1, 6, 7).

Although atenolol has been used instead of propranolol to treat infantile hemangiomas (IH), there is no significant difference in their efficacy (1, 6, 8). According to a meta-analysis, propranolol was more effective in treating hemangiomas than the other treatments (OR = 9.67, 95% CI: 6.62, 14.12, P < 0.001) (9).

## 2. Objectives

In this study, we present the findings of a 5-year prospective descriptive analysis of medical records of infants with hemangiomas who were treated entirely with propranolol (2017 to 2021).

## 3. Methods

We conducted a cross-sectional prospective descriptive study of propranolol-treated pediatric patients with IH from 2017 to 2021. The study was reviewed and approved by the Research Ethics Committee of Shahid Beheshti University of Medical Sciences, Iran (IR.SBMU.MSP.REC.1401.031).

Of the patients who were referred to the clinic for hemangiomas, those who had regular follow-up visits were included.

The primary aim of this study was to evaluate the effect of propranolol treatment on IH, and the secondary aim was to investigate the association of propranolol treatment with the duration of treatment and the age of treatment onset.

Data were collected about new patient visits, general information about the patient, including age and sex, clinical data, and IH management. The study excluded patients who did not receive propranolol or treatment modalities, such as laser therapy. A medical team composed of a pediatrician, a dermatologist, a pediatric surgeon, and a pediatric cardiologist decided on the first referral and indication for propranolol treatment based on the guidelines of the American Academy of Dermatology (10). Parents gave written consent for beginning the treatment with propranolol and for taking serial photos to evaluate the lesion's initial appearance, growth, and evolution during treatment. Detailed patient history was taken, and physical examinations were performed to determine the risk factors for the use of propranolol, such as asthma, reactive airway disease, cardiac arrhythmia, hypoglycemia, and cardiac disease. Patients received propranolol for 1 year. They were treated with propranolol at a dose of 1 mg/kg/day in the first week and 2 mg/kg/day in the second week, and then 3 mg/kg/day was given for the rest of the time. Twenty-four-hour monitoring was performed following the administration of propranolol to infants under 3 months of age or with significant comorbidities. Before beginning the treatment with propranolol (1 mg/kg/day), electrocardiography, blood pressure, and heart rate were recorded. The dosage of propranolol was increased to 3 mg/kg/day, with follow-up visits every 1 - 2 weeks. We met the patients during a series of visits. Case notes and serial digital photos taken at each visit were analyzed to compile the data. The attending physician evaluated the degree of improvement based on clinical assessment compared to the previous photographs.

**Figure 1.**
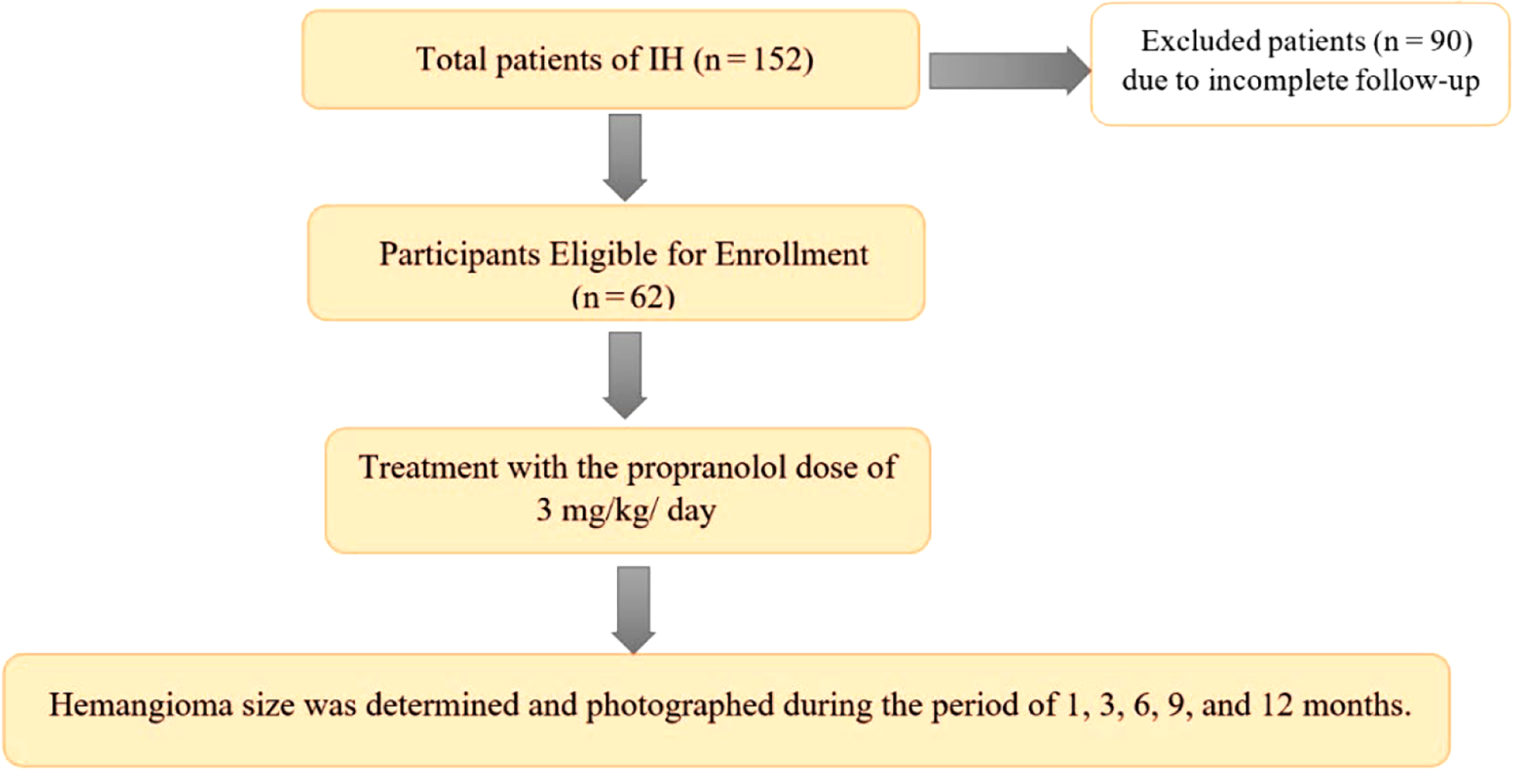
Study diagram

The size, color, and elevation of IH were among the parameters evaluated for improvement. The intervals include the first visit, 1 month, 3 months, 6 months, 9 months, and 12 months after the beginning of the treatment. In every visit, electrocardiogram (ECG) monitoring was performed to avoid propranolol side effects. Approximately 90 cases were excluded from the analysis due to insufficient follow-up (Figure 1). 

The hemangioma score was assessed based on the infantile hemangioma referral score (IHReS) table mentioned in Appendix 1 in the Supplementary File.

### 3.1. Data Analysis

The data were analyzed in SPSS^®^ v. 21 (IBM Corp., Armonk, NY, USA), and the graphs were drawn in Prism GraphPad^®^ v. 8 (GraphPad Software, Boston, MA, USA).

## 4. Results

There were 152 patients referred to the clinic for hemangiomas, but only 62 were included in this study since they had regular follow-up visits.

**Figure 2.**
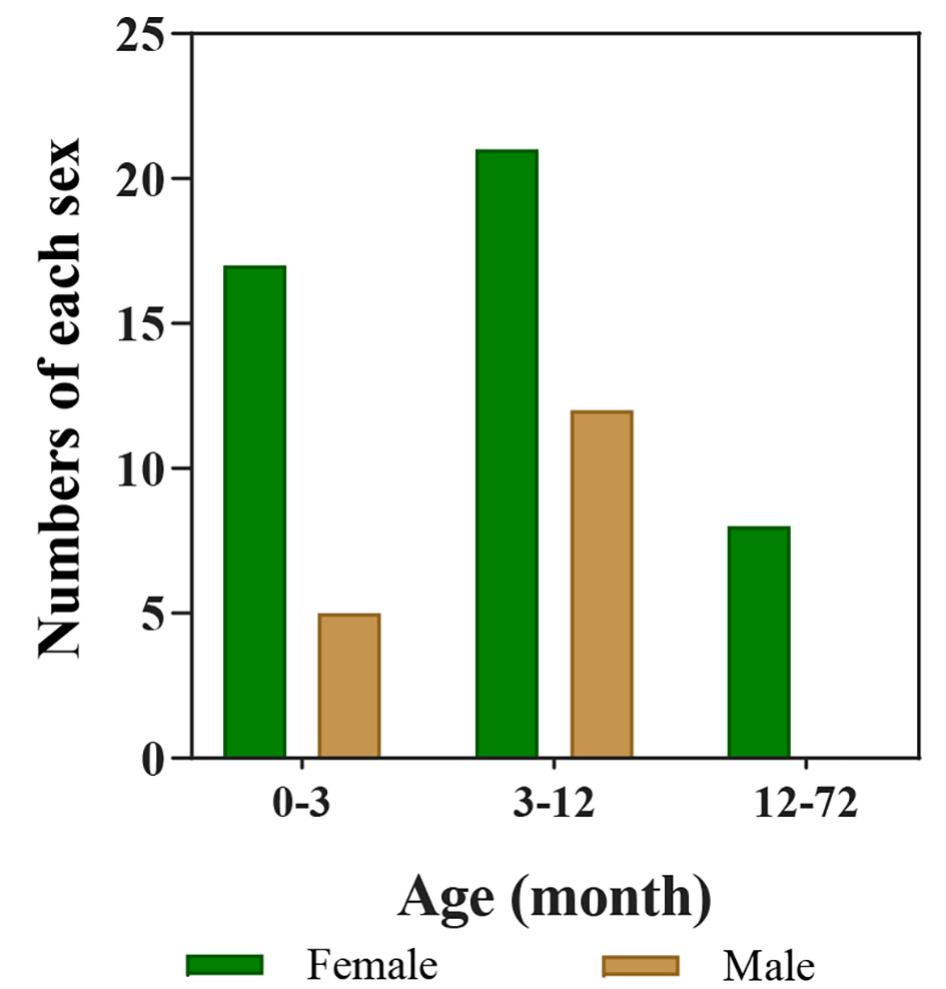
The age distribution of female and male hemangioma patients

**Figure 3.**
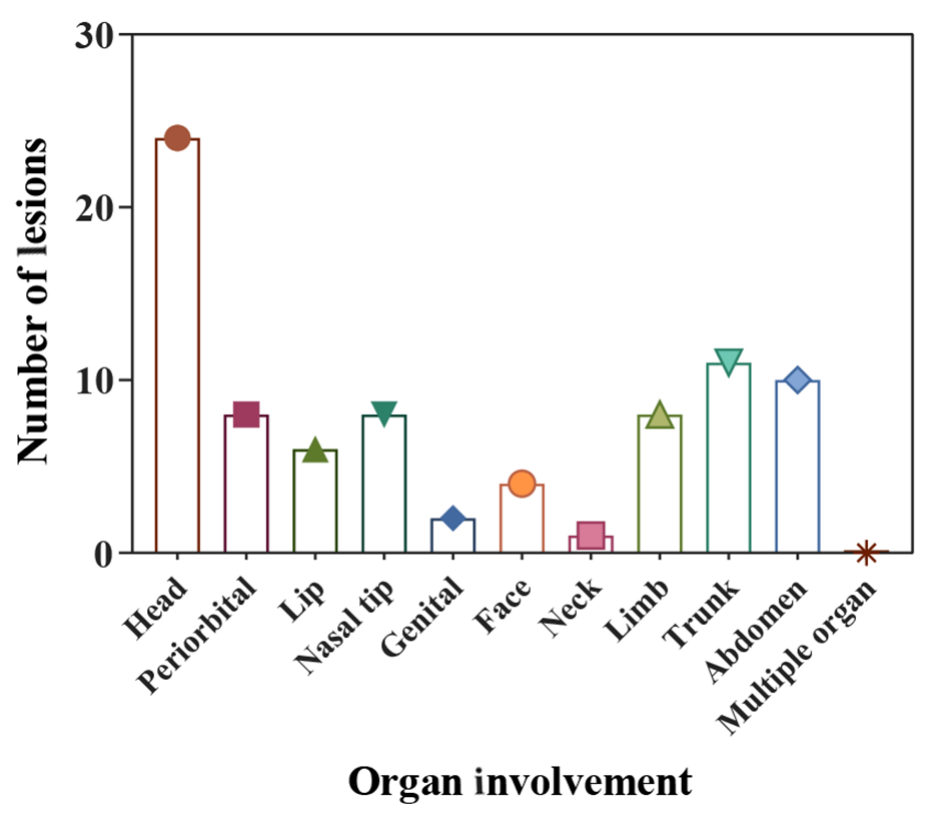
The anatomic location of the hemangiomas

The study included 62 infants, 17 (27.4%) males and 45 (72.6%) females (Figure 2). The analysis revealed no significant sex-related differences (P-value = 0.58). The minimum and maximum ages of the infants at the first visit were 1 month and 60 months, respectively. The weights of the infants at the first visit ranged from 3 to 18 kg, and their hemangioma score ranged from 2 to 6. Based on the number of hemangiomas in the patients, they were divided into three groups. Of these, 46 (74.2%) patients had 1 lesion, 12 (19.4%) had 2 lesions, and 4 (6.5%) had 3 lesions. In another classification, the patients were categorized by the number of lesions in each part of the body, including the scalp, around the eyes, lips, tip of the nose, genitalia, face, neck, limbs, trunk, abdomen, multi-organ involvement, and intravascular type. The most common location was the head (24, 39%), followed by the trunk (11, 17.74%), the abdomen (10, 16.13%), the limb, periorbital, and nasal tip (each with 8 patients, 12.9%), the lip (6, 9.7%), face (4, 6.5%), genitals (2, 3.23%), and neck (1, 1.6%) (Figure 3). Echocardiography was performed on all the patients. There were 58 (93.5%) patients with a normal echo, and 4 (6.5%) had a congenital heart disease (isolated ventricular septal defect (VSD), isolated patent ductus arteriosus (PDA), mild pulmonary valve stenosis (PS), PDA, and VSD). Five patients (9.1%) were completely treated, of whom 1 improved after 3 months, 3 after 6 months, and 1 after 9 months. Besides, 57 (91.9%) patients were partially treated (showed improvement in appearance).

**Figure 4.**
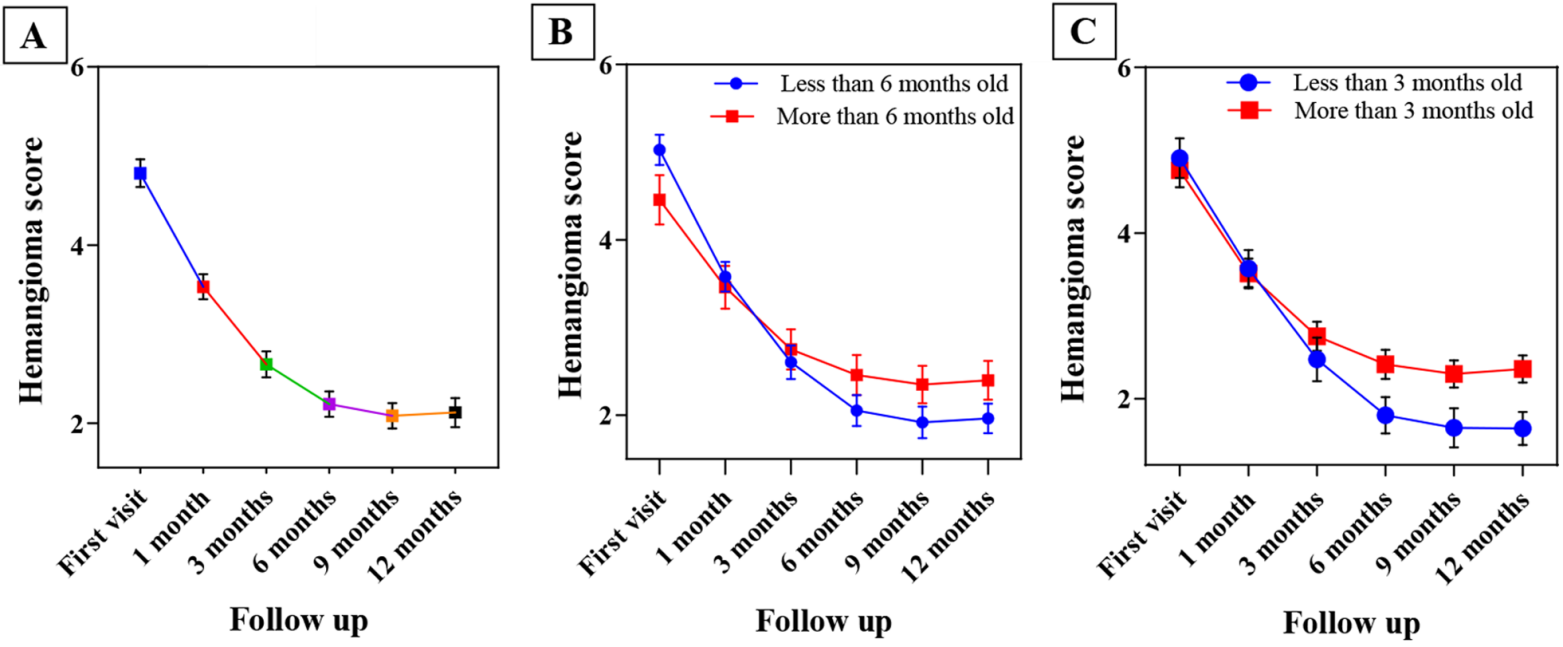
The trend of changes in the mean hemangioma score over the measurement periods (A); less and more than 6 months old (B); less and more than 3 months old (C). Mean ± standard error of the mean (SEM).

Figure 4A shows the trend of changes in the mean hemangioma score over time. A notable observation is that reduction is evident until period 4 (6 months); after this period, the same degree of reduction is not visible, and the potency of treatment effects declines after this point.

The infants were divided into two groups (less than 6 months old and older than 6 months old) for evaluation of the efficacy of the propranolol onset time, and the rate of improvement in these two groups was assessed. In the group aged less than 6 months, 4 cases improved, while 34 cases did not completely improve. In the group aged more than 6 months, only 1 case improved, and 23 cases did not improve. In our analysis, the effect of propranolol on changes in hemangioma size was constant and significant over time, so the average size of hemangiomas was steadily decreasing over time. Therefore, the treatment was effective in reducing the patients’ hemangioma score, and this trend was constant and significant (P-value = 0.000; Figure 4B). Fisher’s exact test results showed that starting treatment at less than 6 months of age had no significant effect on IH treatment (P-value = 0.358).

For further evaluation, the patients were divided into two groups based on the duration of propranolol use: Less than and more than 3 months of propranolol use. Four cases in the group of less than 3 months showed improvement, while 17 cases did not. In the group of more than 3 months, 1 case improved, and 40 cases did not improve. Figure 4C depicts the mean hemangioma score at different times (less than 3 months and more than 3 months). In almost all time periods, the mean hemangioma score in patients who started treatment less than three months was lower than the mean hemangioma score in those who received treatment more than three months. The results of Fisher’s exact test indicated that treatment was significantly more effective in infants who were treated less than three months (P-value = 0.041). The hemangioma score decreased faster in the first months of treatment than in the last months (Figure 4C). Based on Figure 4B and C, the greatest treatment response was observed during the first 3 months of treatment.

There were no adverse effects among our patients.

## 5. Discussion

The safety and efficacy of oral propranolol therapy have been well-documented for a decade since its discovery as a treatment for IH (4, 11, 12). In this paper, we describe infants treated with 3 mg/kg/day propranolol for IH over a 5-year period in a prospective descriptive study.

The study included 62 IH patients between 2017 and 2022. Due to a lack of participation and missed follow-ups, a number of patients were not recorded in this study as in others (4, 13-15).

Of those with IH, 53 patients (85.5%) aged 1 - 10 months old (85%). This value is consistent with the findings of Lydiawati and Zulkarnain, who found that IHs are common in children under the age of 1 year. This condition develops actively during the first few weeks after birth and lasts until it spontaneously involutes around 1 year of age (16).

The majority of IH patients (45, 72.6%) were female and 17 were male (27.4%). Other studies suggest that females are more likely than males to develop IH (3, 4, 14, 15). Epidemiological data also demonstrate a 3-5:1 sex ratio among IH patients (17). According to our study of patients with IH, the ratio of females to males was approximately 2.7:1.

Propranolol is a non-selective β-adrenergic blocker widely used in cardiac disorders. This drug is also used in the treatment of IH (3, 4). Patients with IH may have cardiac disorders. The prevalence of cardiac disorders in IH is reported to be about 0.8% to 7.5% (5, 6). Propranolol administration in IH may be useful for cardiac disorders, but this issue should be investigated in further studies.

Infantile hemangioma was most frequently found in the head region, where 24 patients were affected (39%). Other typical areas included the trunk with 11 infants (17.74%) and the abdomen with 10 infants (16.13 %). It is possible for IH to develop anywhere in the body. Infantile hemangiomas rarely have a negative impact on the child or infant's health, but a few of them, particularly those that develop on the head, face, or neck, can have adverse effects on both appearance and function (18). Many studies have shown that hemangioma lesions most commonly occur in the head and neck regions (11, 13, 18, 19). Our findings were similar to theirs for the head area but less so in the neck area (1, 1.6%).

**Figure 5.**
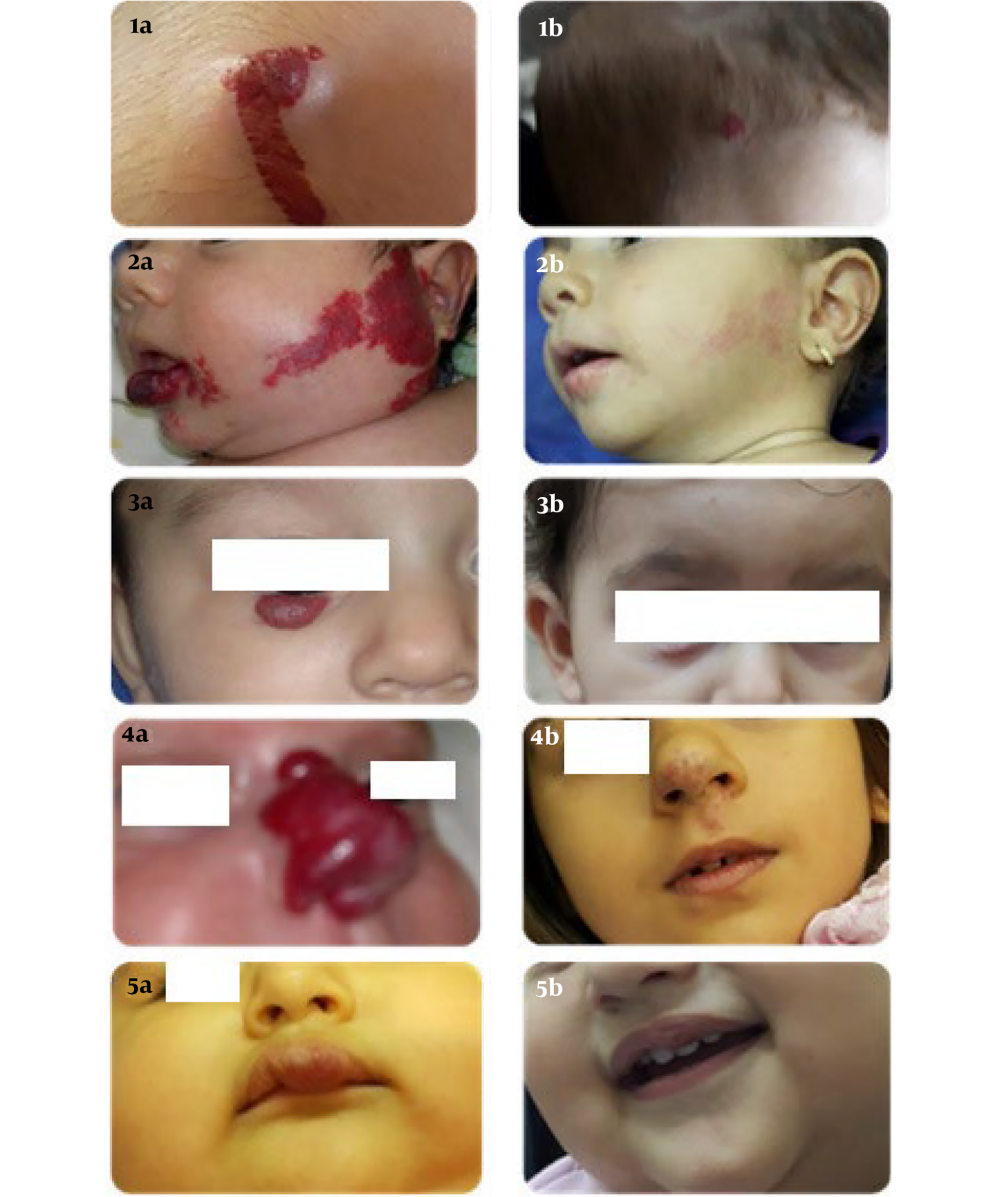
1-5A, before; and 1-5B, after; photos of propranol-treated infants' hemangioma in the scalp, face, around the eyes, nose, and lips, respectively

In this survey, 3 mg/kg/day propranolol demonstrated greater efficacy for the treatment of IHs, especially those initiated under 3 months. Propanol has been found to decrease the size of and lighten IH when given early in the proliferative phase (4, 15). In the study by Oksiuta et al., propranolol therapy at 3 mg/kg resulted in a 72-hour response in 38 patients. Treatment was continued in 39 patients for periods ranging from 1.5 to 17 months. Four patients responded favorably, with more than a 50% reduction in hemangioma size (19). In our analysis, the effect of hemangioma size change was constant and significant over time, so the average size of hemangiomas was steadily decreasing over time. Five patients (9.1%) were completely treated; 1 patient improved after 3 months, 3 after 6 months, 1 after 9 months, and 57 (91.9%) patients were partially treated (Figure 5). 

Based on our findings, it is better to start treatment early in children with IH. Chang et al. mentioned that the age of starting propranolol treatment is not related to the treatment outcome, but a shorter duration of oral propranolol treatment was associated with better results (20). We found that starting propranolol for IH treatment did have an association with the patients' age, a result that contrasted Chang et al.’s finding. We found that a shorter duration of receiving propranolol was associated with better outcomes, which was similar to Chang et al.’s study (20).

Prasad et al. mentioned that propranolol at a lower dose of 1 - 1.5 mg/kg/day is safe and effective in the treatment of IH, and lesions that do not initially respond to the lower dose are unlikely to be treated with a higher dose such as 3 - 4 mg/kg /day (21). These findings were different from our findings. In the current study, the treatment started at a dose of 1 mg/kg/day and reached 3 mg/kg/day, and a good clinical response was observed in all the patients. Besides, in the study by Moyakine et al., it was concluded that the type of IH may play a role in treatment decisions, but in our study, the type of IH did not have a significant effect on the treatment response, and this finding was different between the two cited studies (22).

### 5.1. Conclusions

Infantile hemangioma is the most frequent benign tumor in infancy with unknown pathogenesis. Oral propranolol was found to be more effective in the treatment of hemangiomas, particularly in infants under 3 months of age. Hemangioma was more common in females with head localization. Complications of propranolol treatment were not seen in any of our patients. As a result, our review confirms previous findings that early treatment of IH with propanol is effective and safe.

ijpr-22-1-135140-s001.pdf
