# Supplementary material for: The Efficacy and Safety of Propranolol in Treating Infantile Hemangioma: A Prospective Study
Source: Iran J Pharm Res. 2023 Oct 14;22(1):e135140. doi: 10.5812/ijpr-135140 (PMC12534736; doi:10.5812/ijpr-135140)
Supplement: ijpr-22-1-135140-s001.pdf [file ijpr-22-1-135140-s001.pdf]

Appendix 1.

IHReS

For each parameter, tick **"Yes"** or **"No."**

The total score is the sum of the scores from each parameter below.

Parameters	Items			Score<br>Please consider only the highest score for each parameter
**Location of hemangioma**	Other facial areas than those mentioned previously	◯ Yes ◯ No	If yes: 3 points<br>*(if no: 0 point)*	◯3 ◯2 ◯0
	Neck, diaper area, scalp	◯ Yes ◯ No	If yes: 2 points<br>*(if no: 0 point)*	
**Size of the biggest hemangioma**	≥1 cm on other facial area than those mentioned previously	◯ Yes ◯ No	If yes: 3 points<br>*(if no: 0 point)*	◯3 ◯2 ◯0
	2 to 4 cm on other body area than those mentioned previously	◯ Yes ◯ No	If yes: 2 points<br>*(if no: 0 point)*	
**Current child age and growth of hemangioma**	The infant is <2 months	◯ Yes ◯ No	If yes: 3 points<br>*(if no: 0 point)*	◯3 ◯2 ◯0
	The infant is ≥2 and ≤4 months, with an evident growth within the last 2 weeks	◯ Yes ◯ No	If yes: 2 points<br>*(if no: 0 point)*	
			**Total**	

Score ≥4: please refer the patient to an expert center.
Score <4: the patient is not to be referred and should be monitored. The score will be done at every visit.

The final decision to refer the patient to an expert centre is up to the physiciab and the parents.
